# Hand position-dependent modulation of errors in vibrotactile temporal order judgments: the effects of transcranial magnetic stimulation to the human posterior parietal cortex

**DOI:** 10.1007/s00221-014-3861-9

**Published:** 2014-02-22

**Authors:** Anina Ritterband-Rosenbaum, Robert Hermosillo, Gregory Kroliczak, Paul van Donkelaar

**Affiliations:** 1 Department of Nutrition, Exercise and Sports, Panum Institute, University of Copenhagen, Copenhagen, Denmark; 2 Department of Neuroscience and Pharmacology, Panum Institute, University of Copenhagen, Copenhagen, Denmark; 3 School of Health and Exercise Sciences, University of British Columbia, Kelowna, Canada; 4 Action and Cognition Laboratory, Institute of Psychology, Adam Mickiewicz University in Poznań, Poznan, Poland

**Keywords:** Temporal order judgment, Transcranial magnetic stimulation, Posterior parietal cortex, Vibrotactile, Spatial

## Abstract

The ability to decide which of the two stimuli is presented first can be probed using a temporal order judgment (TOJ) task. When the stimuli are delivered to the fingers, TOJ decisions can be confounded by the fact that the hands can be moved to different locations in space. How and where this confounded information is processed in the brain is poorly understood. In the present set of experiments, we addressed this knowledge gap by using single-pulse transcranial magnetic stimulation (TMS) to disrupt processing in the right or left posterior parietal cortex (PPC) during a vibrotactile TOJ task with stimuli applied to the right and left index fingers. In the first experiment, participants held their hands in an uncrossed configuration, and we found that when the index finger contralateral to the site of TMS was stimulated first, there was a significant increase in TOJ errors. This increase did not occur when stimuli were delivered to the ipsilateral finger first. In the second experiment, participants held their hands in a crossed configuration and the pattern of errors was reversed relative to the first experiment. In both the first two experiments, significant increases in TOJ error were present with TMS over either hemisphere, regardless of arm configuration; however, they were larger overall following TMS over the right PPC. Control experiments using sham TMS indicated the systematic modulation in error was not due to nonspecific effects of the stimulation. Additionally, we showed that these TMS-induced changes in TOJ errors were not due to a reduced ability to detect the timing of the vibrotactile stimuli. Taken together, these results demonstrate that both the right and left PPC contribute to the processing underlying vibrotactile TOJs by integrating vibrotactile information and proprioceptive information related to arm position in space.

## Introduction

A vital aspect of human behavior is the ability to keep track of time. Despite the clear need for temporal discrimination, the neural mechanisms underlying time processing are poorly understood. In laboratory settings, temporal processing can be probed using temporal order judgment (TOJ) tasks. In such tasks, two stimuli are presented in quick succession, typically in the visual or auditory domain, and the participant makes a decision as to which arrived first. Such tasks are simple when the interstimulus interval (ISI) is long in duration, but become increasingly difficult as the ISI approaches zero.

Unlike with stimuli presented in the visual or auditory domain, when vibrotactile stimuli are used in a TOJ task, it is possible to alter their spatial location by changing the configuration of the limbs, for example, by applying the stimuli to each index finger and varying the relative position of the hands with respect to the midline of the body. Yamamoto and Kitazawa (2001) demonstrated that when the hands are uncrossed, decisions regarding the temporal order of such stimuli are accurate even with ISIs as short as 70 ms; however, with the hands in a crossed configuration, ISIs of up to 600 ms are required to make accurate judgments. They suggested that this deficit is due to the additional time necessary to resolve the relative spatial location of the stimuli with the hands in the crossed configuration. By contrast, Shore et al. (2002) have hypothesized that the conflict induced by the spatial mismatch between anatomical and allocentric reference frames leads to the deficit when the arms are crossed. Common to both of these accounts is the spatial remapping required to successfully perform the task.

A number of brain imaging studies have examined the cortical sites at which this spatial remapping is carried out in the context of a vibrotactile TOJ task. Each of these studies demonstrated that the posterior parietal cortex (PPC) showed greater activity associated with the remapping process when the arms were in a crossed posture (Lloyd et al. 2003; Takahashi et al. 2013; Wada et al. 2012). The current study sought to determine the functional necessity of the PPC involvement by using single-pulse transcranial magnetic stimulation (TMS) to temporarily disrupt neural processing in either the right or left PPC, while participants performed the vibrotactile TOJ task with the hands in either an uncrossed or crossed configuration. We hypothesized that if the PPC contributes to the remapping process on which TOJ decisions depend, then disrupting the PPC with TMS should systematically modulate TOJ errors in a manner which is dependent on the crossed or uncrossed configuration of the hands.

## Materials and methods

### Participants

Fifty-one naïve healthy right-handed subjects and 2 healthy left-handed subjects (25 female, 28 male) aged 19–35 years (mean age = 23.4 ± 4.0 year) participated in the experiment. Exclusion criteria included no previous history of sensorimotor deficits or personal or family history of seizure. All participants signed an informed consent form which explained the nature of the procedure and the small but potential risks of the application of TMS. The research was approved by the Committee for the Protection of Human Subjects at the University of Oregon and the Behavioural Ethics Research Board at the University of British Columbia and was conducted in accordance with the Declaration of Helsinki guidelines. Participants were paid $10 for each session in which they participated.

### Experimental apparatus

The participant sat comfortably in a chair with their eyes open and their arms resting palms down ~15 cm apart on a table. A piezoelectric vibrotactile stimulator was attached to the underside of the distal segment of the extended index finger pad of each hand with Velcro straps. Stimulation was achieved by applying a single rectangular voltage pulse (5 V, 5 ms) to the piezoelectric device producing a small displacement (1 mm) of the contact point (2 mm^2^) so as to touch the surface of the skin.

### Experimental task

**Fig. 1:**
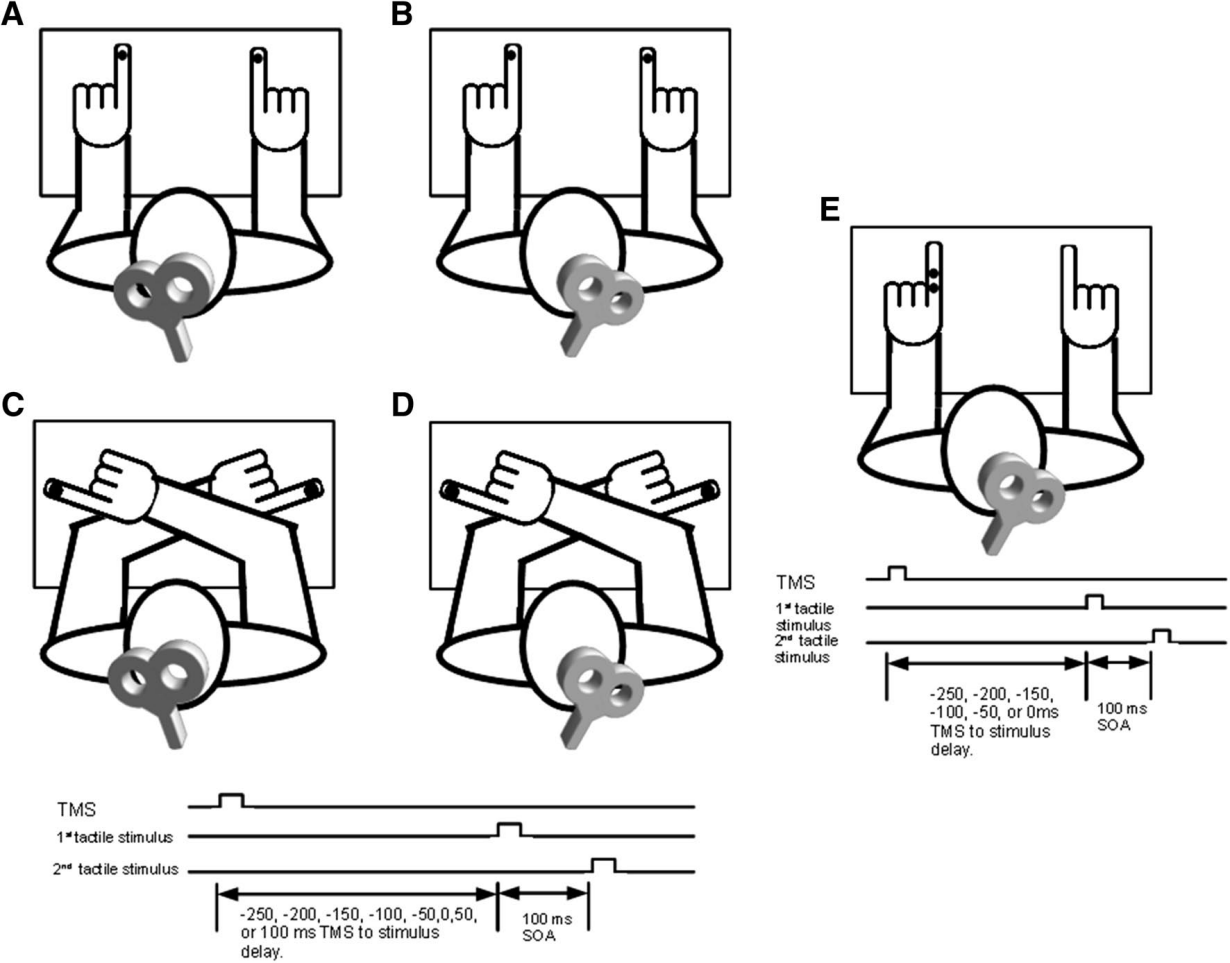
Experimental setup—spatial TOJ task—vibrotactile hand stimulation was delivered to the index finger of each hand with an ISI of 100 ms. On 67 % of the trials, a single pulse of TMS was delivered over the PPC 250, 200, 150, 100, 50 ms prior to, coincident with, or 50 or 100 ms after, the initial hand was stimulated. In all trials, both hands were stimulated. In separate sessions, real or sham TMS was delivered to the right or left PPC, and for any of these combinations, participants performed the task with either the arms crossed or uncrossed. For experiments 1 and 3, participants used configurations **a** and **b**. For experiments 2 and 4, participants used configurations **c** and **d**. Experiment 5—vibrotactile hand stimulation was delivered to the middle and proximal pads of the left index finger with an ISI of 100 ms are shown in **e**. On 85.7 % of the trials, a single pulse of TMS was delivered over the right PPC 250, 200, 150, 100, 50 ms prior to, or coincident with the initial finger pad stimulation. In all trials, both the middle and proximal pads were stimulated. For this control experiment, participants performed the task with the arms uncrossed

On each trial, the participant received successive vibrotactile stimulation to each index finger separated by an interstimulus interval (ISI) of 100 ms (Fig. 1). This ISI has been shown to result in an increase in TOJ error rates above baseline but below chance levels (Shore et al. 2002; Hermosillo et al. 2011) when the arms are uncrossed. On half the trials, the right index finger was stimulated first, whereas on the other half, the left index finger was stimulated first. After the stimulation to both fingers, the participant was required to make an unspeeded, forced-choice verbal TOJ indicating which finger (“right” or “left”) was stimulated first. We chose to use a verbal report to prevent confusion that may have been present had manual responses been used instead. All responses were recorded by the experimenter.

### Transcranial magnetic stimulation

A 2T Magstim Mono pulse 200 was used to deliver single TMS pulses via a figure eight coil. Stimulation was delivered at 110 % of the resting motor threshold for reliably eliciting an observable twitch of the first dorsal interosseus (FDI) of the contralateral hand at the motor hot point. The motor threshold from the right hemisphere M1 was used to calculate the stimulation intensity for the right PPC and that of the left M1 was used for the left PPC. During each experimental condition, the stimulating coil was moved 7 cm posterior to the motor hot point to a position that was an average of 17.44 ± 5.1 mm superior and 6.72 ± 1.96 mm anterior to the P3 and P4 sites in the international 10–20 EEG system (Herwig et al. 2003).

**Fig. 2:**
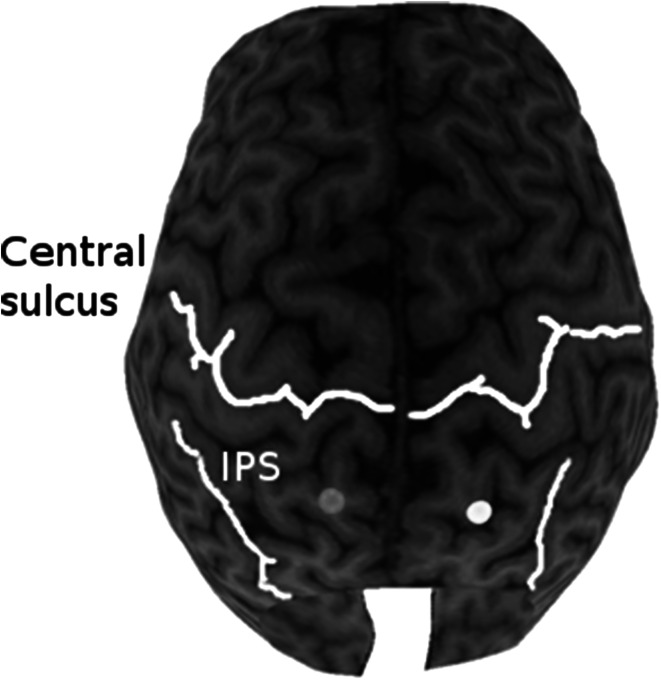
Reconstructed PPC stimulation sites—reconstructed stimulation sites for a single participant in the left (*red*) and right (*yellow*) superior parietal lobule. The central sulcus and intraparietal sulcus are indicated for reference (color figure online)

The PPC TMS sites were confirmed in one participant using the Brainsight system (Rouge Research Inc, Montreal, Canada). For this purpose, a whole-brain anatomical scan was obtained from the participant using a T1-weighted magnetization-prepared rapid gradient-echo sequence (time repetition = 2,500 ms, echo time = 4.38 ms, flip angle = 8^o^, field of view = 256 × 256 mm; 176 slices per slab at 1 mm slice thickness). Figure 2 shows the resulting reconstructed stimulation sites over the superior parietal lobule in the left and right PPC. These sites are in close proximity to regions shown to be activated by the behavioral task (Takahashi et al. 2013) and to the sites used in several TMS studies in which the PPC was targeted (e.g., Della-Maggiore et al. 2004; Vesia et al. 2008).

During the task, participants wore a swim cap on which markings were made to facilitate TMS placement. The coil handle faced backwards at a 45° angle from the midline, and the coil and head were stabilized with a clamping system and chin rest, respectively. None of the subjects reported any undesirable side effects resulting from the stimulation.

### Experimental conditions

We performed 5 experiments to investigate how the configuration of the arms and how TMS to specific hemispheres affected the TOJ decisions:

In Experiment 1, we targeted either the left or the right PPC with TMS, while the participant had his or her arms uncrossed (Fig. 1a, b).

Experiment 2 was the same as Experiment 1 except the participant had his or her arms in a crossed configuration (Fig. 1c, d).

Experiments 3 and 4 were analogous to experiments 1 and 2, respectively, except that subjects received sham TMS over the PPC rather than real TMS. Sham TMS was achieved by turning the stimulating coil around such that the TMS pulse was directed away from the skull. We decided to employ Sham TMS instead of using an alternative stimulation site because we felt it most closely controlled for the potentially confounding and nonspecific effects of the real TMS on task performance.

Within each of the first 4 experiments, the arm configuration (crossed vs. uncrossed), hemisphere stimulated (right vs. left), and type of TMS stimulation (real vs. sham) were held constant. Subjects had full vision of his or her hands in each experiment, and the room was illuminated. By keeping each of these aspects constant, we effectively controlled for the known influence of vision on TOJ errors (Lloyd et al. 2003).

Within each of these experiments, TMS was delivered 250, 200, 150, 100, or 50 ms prior to, coincident with, or 50 or 100 ms after the first finger stimulation (see Fig. 1). The participant completed 10 blocks comprised of 18 trials, 9 with either the right or left hand stimulated first with each of the 8 TMS delays and 1 additional trial without TMS which were used as a baseline for a total of 180 trials per participant. Each trial lasted approximately 10 s including the verbal response, and the complete experiment took approximately 45 min including short breaks in between the blocks. The different combinations of trial types were pseudorandomly interleaved such that the participant was never aware which hand was going to be stimulated first, or whether or when they would receive TMS.

In experiment 5, we applied TMS over the right PPC while the subject received two vibrotactile stimuli on the left hand (Fig. 1e). This allowed us to directly assess whether PPC encodes the timing of vibrotactile signals. The stimulators were attached to the proximal and middle pad of the left index finger and on half the trials the middle pad was stimulated first, whereas on the other half, the proximal pad was stimulated first. Subjects were required to make an unspeeded decision regarding which stimulus was felt first with the forced-choice options of “closer” or “farther.” All responses were recorded by the experimenter. TMS was delivered over the right PPC 250, 200, 150, 100, 50 ms prior to, or coincident with the first stimulation of the finger (Fig. 1e). The participant completed 1 block comprised of 140 trials, 10 with either the middle or proximal index finger pad stimulated first with each of the 6 TMS delays and 20 additional baseline trials without TMS.

### Data analysis

Our measure of interest was the percentage of responses that were incorrect. This was calculated for each combination of TMS delay and first stimulation site and normalized to the error rate for the trials without TMS for all subjects by subtracting the non-TMS trial error. Error rates were normalized in this way because it has been well documented that crossing the arms reduces TOJ accuracy (Shore et al. 2002; Yamamoto and Kitazawa 2001; Azañón and Soto-Faraco 2007; Schicke and Röder 2006; Yamamoto et al. 2005). Experiments 1 through 4 were all analyzed using separate 2 (hand stimulated first: left vs. right) × 2 (hemisphere: left vs. right) × 8 (TMS delay: −250, −200, −150, −100, −50, 0, 50, or 100 ms) 3-way repeated measures ANOVAs with hand stimulated first and TMS delay as within-subject factors. Experiment 5 was analyzed with a 2 (stimulation site) × 6 (TMS delay: −250, −200, −150, −100, −50, 0 ms) 2-way repeated measures ANOVA with stimulation site and TMS delay as within-subject factors. In each case, eight (or six) pairwise post hoc multiple comparisons were completed with a Bonferroni-corrected alpha level set at 0.05 to examine differences within each of the different TMS delays.

## Results

**Fig. 3:**
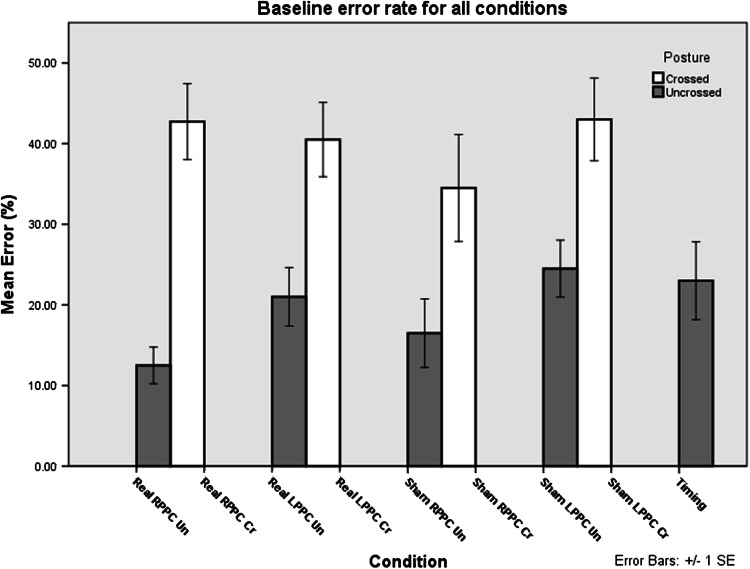
Baseline error rate of non-TMS trials—Group means for the error rates in the trials without TMS across all conditions. Uncrossed postures are shown in *gray*, and crossed postures are shown in *white*. *Error bars*, 1 intersubject SE

We were interested in the extent to which TMS over the PPC could disrupt the processing associated with TOJ decisions and whether this would be modulated by the spatial configuration of the hands. Error rate was the dependent variable of interest, and it was normalized with respect to the non-TMS baseline trials that were intermingled with the TMS trials. Initial analysis of non-TMS trials indicated that normalized error rate was greater with the hands in the crossed (40.50 %) compared to uncrossed (18.63 %) configuration (*t*(158) = 4.942, *p* < 0.001) as has been demonstrated previously (Fig. 3, white bars vs. gray bars excluding experiment 5) (Yamamoto and Kitazawa 2001; Shore et al. 2002; Hermosillo et al. 2011).

### Experiment 1: real TMS: arms uncrossed

**Fig. 4:**
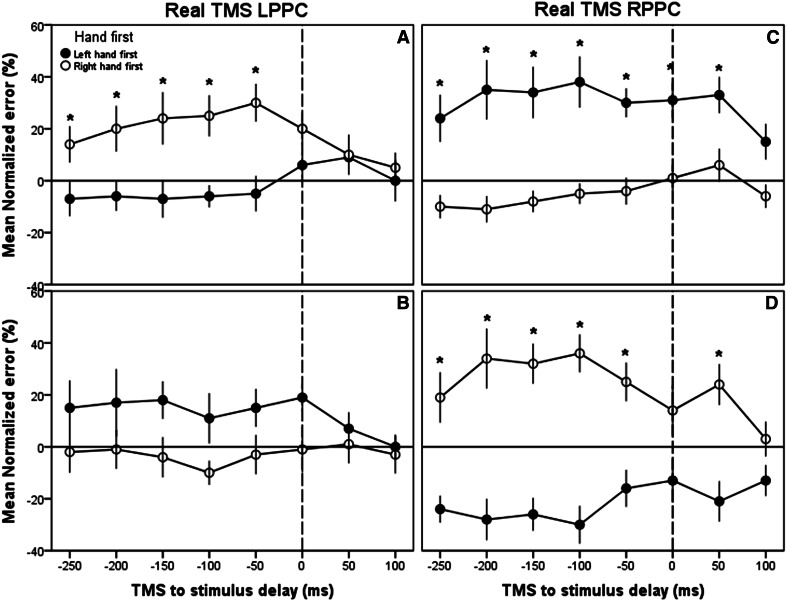
Experiments 1 and 2: effects of PPC TMS on TOJ decisions—Group means for normalized error rate across the different TMS delays. Data are shown separately for the *left* (**a**) and *right* (**b**) PPC TMS with the arms in the uncrossed configuration (*top*) or for *left* (**c**) and *right* (**d**) PPC TMS with the arms in the crossed configuration (*bottom*). The *dotted lines* represent the time for the firsthand stimulation. *Black circles*, left hand stimulated first; *white circles*, right hand stimulated first. *Asterisks*, *p* < .05 across hands. *Error bars* 1 intersubject SE. *Horizontal line* indicates no change in error when compared to no-TMS trials

In experiment 1, we observed no significant main effect for the hand that was stimulated first (*F* [1,18] = 1.531, *p* = n.s.) or hemisphere to which TMS was applied (*F*[1,18] = 0.371, *p* = n.s); however, there was a main effect of delay (*F*[7,126] = 2.780, *p* < 0.05, partial *η* ^2^ = 0.134). Additionally, there was a significant interaction between the PPC hemisphere stimulated and the hand stimulated first (*F*[1,18] = 23.324, *p* < 0.001, partial *η* ^2^ = 0.564), such that TMS applied to the left PPC led to significantly higher normalized error rates when the right hand was stimulated first relative to when the left hand was stimulated first (−2.0 ± 5.60 SE vs. 18.5 ± 4.95, respectively). This pattern was reversed when TMS was applied to the right PPC, such that in left-first trials, there was a significantly higher normalized error rate than in right-first trials (−4.5 ± 4.95 vs. 30.0 ± 5.60, respectively). All the above-mentioned results should be interpreted with caution because we also observed a quite intuitive significant 3-way interaction between hand stimulated first, TMS delay, and the hemisphere to which TMS was applied (*F*[7,126] = 4.507, *p* < 0.001, partial *η* ^2^ = 0.200). To facilitate the understanding of these results, this interaction was broken down and further analyzed with Bonferroni-corrected pairwise post hoc comparisons within each of the combinations of conditions represented in the panels in Fig. 4. The results of these comparisons demonstrated that there were significant differences between normalized error rates across the hands at all the delays except at +100 ms in the right PPC condition and at the −250 to −50 ms delays in the left PPC condition, implying that the effects of TMS were greater during the period leading up to the first finger stimulation.

### Experiment 2: real TMS: arms crossed

**Fig. 5:**
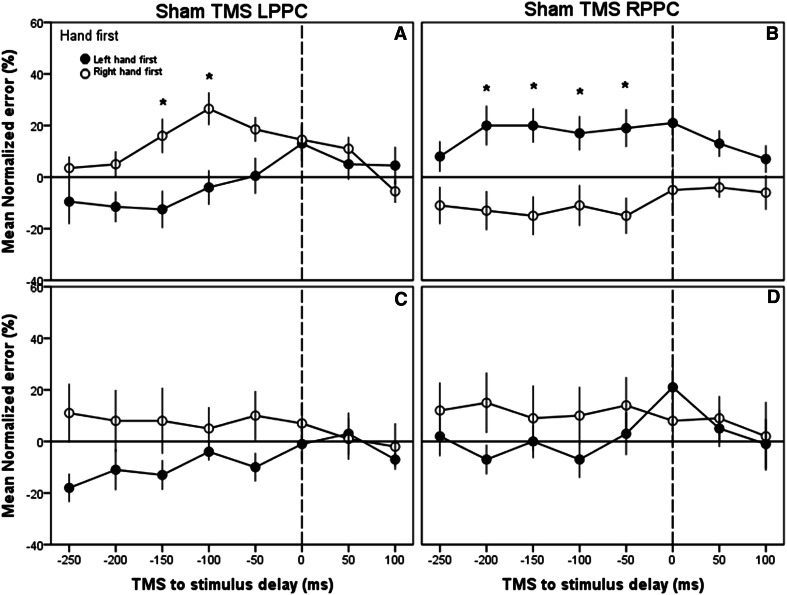
Experiments 3 and 4: control experiment using sham TMS over the PPC—Group means for normalized error rate across the different sham TMS delays. Data are shown separately for the *left* (**a**) and *right* (**b**) PPC sham TMS with the arms in the uncrossed configuration (*top*) or for *left* (**c**) and *right* (**d**) PPC sham TMS with the arms in the crossed configuration (*bottom*). The *dotted lines* represent the time for the first hand stimulation. *Black circles*, left hand stimulated first; *white circles*, right hand stimulated first. *Asterisks* *p* < .05 across hands. *Error bars* 1 intersubject SE. *Horizontal line* no change in error when compared to no-TMS trials

In experiment 2, there were no significant main effects of the hand stimulated first (*F*[1,18] = 3.076, n.s), hemisphere stimulated (*F*[1,18] = 0.81, n.s), or delay (*F*[7,126] = 1.1719, n.s); however, several significant interactions were present. In particular, we observed significant 2-way interactions between the hand stimulated first and the hemisphere to which TMS was applied (*F*[1,18] = 13.218, *p* < 0.01, partial *η* ^2^ = 0.423) and between the hand stimulated first and TMS delay (*F*[7,126] = 2.121, *p* < 0.05 partial *η* ^2^ = 0.105). As in Experiment 1, there was also a significant 3-way interaction between the hand stimulated first, TMS delay, and the hemisphere to which TMS was applied (*F*[7,126] = 5.186, *p* < 0.001, partial *η* ^2^ = 0.224). Bonferroni-corrected pairwise post hoc comparisons revealed that, similar to Exp. 1, there were significant differences between the hands at all the delays except +100 ms and also 0 ms in the right PPC condition. Counter to Exp. 1, the effect was absent in any of the delays in the left PPC condition. This is consistent with the fact that the effects of TMS were greater during the period leading up to the first finger stimulation and were larger overall in the right compared to left PPC (Figs. 4, 5).

### Experiment 3: sham TMS: arms uncrossed

In experiments 1 and 2, we noted a significant effect of delay, and it is possible that these effects were due partially or entirely to nonspecific effects of the TMS drawing attention to the ipsilateral side of space and, thus, increasing the likelihood that the participant would incorrectly perceive the ipsilateral hand being stimulated first when this was not the case. The sham TMS condition allowed us to examine this issue in detail.

In experiment 3, we observed a significant effect of sham TMS delay (*F*[7,126] = 4.247, *p* < 0.01, partial *η* ^2^ = 0.191 (Greenhouse-Geisser)). Nevertheless, there was also a significant 3-way interaction between the hand stimulated first, sham TMS delay, and the hemisphere given sham stimulation (*F*[7,126], *p* < 0.001, $\eta_{\text{p}}^{2} = 0. 2 6 7$, (Greenhouse-Geisser)). No other significant effects were observed. Post hoc tests revealed a significant effect of sham TMS over the left PPC at −150 and −100 ms prior to the firsthand stimulation; however, no other times points were significant. These tests also revealed a significant effect of sham TMS over the right PPC at −200, −150 ms, −100 ms, and −50 prior to the firsthand stimulation.

### Experiment 4: sham TMS: arms crossed

In experiment 4, we observed a significant effect of sham TMS delay (*F*[7,126] = 2.224, *p* < 0.05, $\eta_{\text{p}}^{2} = 0. 1 10$), and a significant interaction between sham TMS delay and the hand stimulated first (*F*[7,126] = 2.225, *P* < 0.05, $\eta_{\text{p}}^{2} = 0. 1 10$). There were no other significant main effects or interactions.

Although these significant interactions were similar to those observed in the real TMS condition, importantly, there were no interactions based on TMS delay and no significant pairwise effects at any of the delays. Taken together, the pattern of results from the sham TMS condition implies that the effects observed for real TMS during the period prior to finger stimulation were due to a specific disruption to the processing associated with the TOJ task in the PPC at these critical times.

A subsequent 5-way ANOVA allowed us to directly compare the impact of real versus sham TMS on this task and revealed a significant main effect of stimulation type (*F*[1,1152] = 6.091, *p* < 0.01), demonstrating that real TMS had a larger impact on TOJ decisions than sham TMS. Furthermore, a significant interaction was observed between hemisphere, hand stimulated first, and arm configuration (*F*[1,576] = 238.79, *p* < .001). This interaction captures the fact that the effects of TMS over the right PPC on normalized error rate were more substantial overall than TMS over the left PPC, and this effect was modulated by the arm configuration and the hand stimulated first. Moreover, the effect sizes (partial *η* ^2^) were larger for real TMS compared to sham TMS.

### Experiment 5: real TMS; proximal versus distal stimulation

**Fig. 6:**
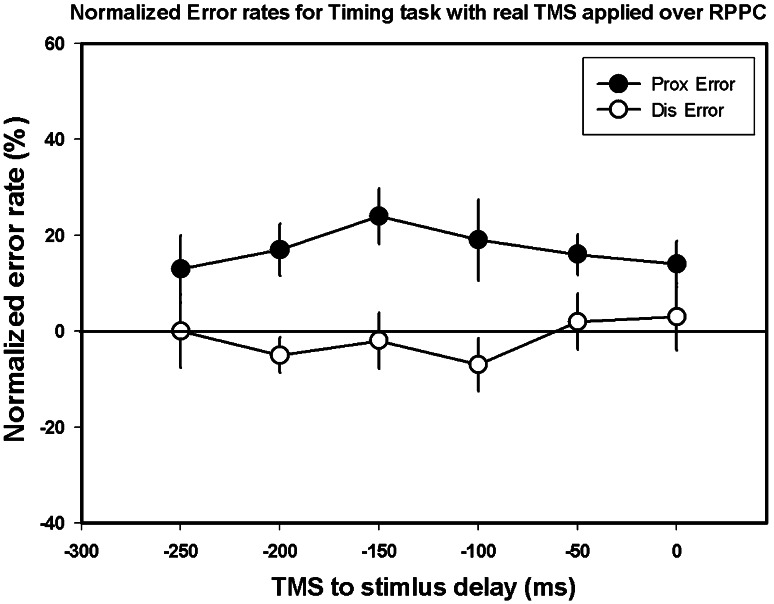
Experiment 5: control timing experiment using TMS over the right PPC—Group means for normalized error rate for “Proximal First” and “Distal First” trials across the different TMS delays. *Black circles* proximal hand stimulated first; *white circles*, distal hand stimulated first. *Error bars* 1 intersubject SE. *Horizontal line* no change in error when compared to no-TMS trials. *Dis* distal, *Prox* proximal

In experiment 5, we examined whether the right PPC contributes more directly to timing processes by characterizing the effects of TMS on the ability to determine the timing of two vibrotactile stimuli delivered to the same finger. Results indicated no significant effect of TMS delay (*F*[5, 108] = 0.218, *p* > 0.05) but a significant difference between the distal and proximal stimulation (*F*[1,108] = 28.07, *p* < 0.001), possibly suggesting a difference in sensitivity of the regions stimulated (Fig. 6). Additionally, there was no significant interaction between the stimulation site and the time at which TMS was applied (*F*[5,108] = 0.621, *p* > 0.05). The results from this control experiment suggest that the PPC does not play a direct role in timing perception at least in the context of TOJ decisions.

## Discussion

The purpose of the present study was to better understand the contribution of the PPC to the processing underlying vibrotactile TOJ tasks. In addition, we were interested in the potential interaction between vibrotactile TOJ processes and the spatial localization of tactile stimuli. In particular, because the hands can move with respect to the midline of the body, it is possible to dissociate the hand from the side of space which is stimulated first (e.g., the left hand getting stimulated first while on the right side of space and vice versa). Yamamoto and Kitazawa (2001) took advantage of this and demonstrated that TOJ errors increase substantially when the hands are crossed. It has been suggested that this is a more challenging context in which to perform a TOJ task because of the spatial remapping required with the hands in a crossed configuration. How the PPC contributed to this spatial remapping process and whether the left and right PPC did so differently were questions that were also considered in the current study.

Our results demonstrated that TMS over either the right or left PPC caused marked increases in TOJ errors when the hand contralateral to the TMS site was the first to receive vibrotactile stimulation, but that this effect was greater in the right PPC. By contrast, error rates were relatively unaffected when the hand ipsilateral to the TMS site was stimulated first. The only exception to this was when TMS was delivered to the right PPC with the arms in a crossed configuration and the left hand (in the ipsilateral side of space) was stimulated first. Under these circumstances, the error rates were actually *reduced* relative to the non-TMS control trials. Thus, under this combination of conditions, participants’ performance improved with the TMS, suggesting that the processing underlying the detection of the initial vibrotactile stimulus to the left hand was more effective. It is unclear why this occurred; however, it could be due to at least two factors: (1) the use of a forced-choice task—participants that perceive stimulation arriving more frequently on one side during TMS are less likely to choose that side on non-TMS trials; or (2) a TMS-induced increase in sensitivity to the ipsilateral hand (Seyal et al. 1995) under these circumstances.

The asymmetry between left and right PPC contributions to TOJ processing was apparent in the range of TMS delays. With right PPC TMS, differences were observed across a much broader range of delays prior to the hand stimulation than with left PPC TMS. By contrast, when the TMS arrived coincident with or in between the two vibrotactile stimuli, the differences across hands were markedly reduced. We suggest that this pattern of results is consistent with the critical involvement of the right, and to a lesser extent, the left PPC in the spatial remapping required to successfully perform the TOJ task (Shore et al. 2002; Yamamoto and Kitazawa 2001; Schicke and Röder 2006). When this remapping is disrupted with TMS, participants have difficulty determining where the vibrotactile stimulation is being delivered.

The relative specificity of the right versus left PPC in the broad context of spatial remapping and the multisensory representation of limb position has been the subject of several recent studies. In particular, it has been reported that tactile stimulation of the right hand, across the body midline, engages the right posterior parietal cortex including VIP when the eyes are closed (Lloyd et al. 2003). In addition, delivering TMS to the right PPC disrupts a task requiring remapping tactile stimulation onto external space (Azañón et al. 2010). By contrast, at least one other study demonstrated greater involvement of the left PPC in the arm crossed configuration, the magnitude of which was correlated with subjective reversals in the TOJ task (Wada et al., 2012). Thus, the specificity of the right versus left PPC in the processes underlying TOJ performance remains to be more fully clarified.

The fact that TMS affected TOJ performance when it was delivered prior to the vibrotactile stimulation in the current study implies that the pattern of PPC activity is critical prior to the actual arrival of the stimuli. This result contrasts with that of at least two previous TMS studies which have investigated the role of the PPC in the detection and discrimination of vibrotactile stimulation outside of the TOJ context. In particular, these studies demonstrated that TMS disrupts vibrotactile detection and discrimination when it is delivered after the stimulation rather than before it (Oliveri et al. 1999; Porro et al. 2007). When compared to the results of the current study, this reinforces the idea that the PPC makes different context-dependent contributions that vary as a function of time.

The context-dependent contribution of the PPC to the TOJ task was further illustrated in Experiment 5. In particular, in this experiment, we examined the explicit contribution of the PPC to the accurate perception of timing within a TOJ task independent of any spatial manipulation. The results showed that TOJ errors were substantially reduced under these circumstances relative to those observed in the 2nd experiment with stimuli applied to each hand in a crossed configuration. Taken together, these results imply that the PPC is directly engaged in the spatial remapping required to perform TOJ decisions when the hands are crossed.

The use of a somatotopic (i.e., indicating which finger was stimulated first) as opposed to an allocentric response (i.e., indicating which side of space was stimulated first) has the potential to allow participants to explicitly avoid remapping in their decisions. In other words, in the crossed con in particular, participants may ignore the allocentric information and focus instead on the somatotopic signals to drive their decisions. While this possibility cannot be completely discounted, we suggest that it is the interaction between the somatotopic information signaling the configuration of the arm and the vibrotactile stimulation that requires the spatial remapping to which the PPC appears to be vital in the current study. It is also important to keep in mind that this mode of responding has been used in most of the previous studies that have investigated the behavioral and neural characteristics of this issue (Hermosillo et al. 2011; Lloyd et al. 2003; Shore et al. 2002; Takahashi et al. 2013; Wada et al. 2012; Yamamoto and Kitazawa, 2001). Thus, to be consistent with these studies and allow the most direct basis for comparison, we chose to maintain somatotopic responses.

In conclusion, we have shown that the human PPC plays a prominent role in vibrotactile TOJ tasks especially in the context of the spatial remapping required when the hands are in a crossed configuration. Furthermore, the right PPC appears to play a more substantial role than the left PPC in the processing underlying TOJ tasks when assessed across a wide time period prior to the vibrotactile stimulation. Taken together, this evidence provides fresh insight into the neural mechanisms underlying spatiotemporal processing and how it interacts with different bodily configurations.

## Abbreviations

TOJ: Temporal order judgment
TMS: Transcranial magnetic stimulation
PPC: Posterior parietal cortex
ISI: Interstimulation interval
